# Posttraumatic Pseudoarthrosis of a Clavicle Fracture in an 11-Year-Old Girl: A Case Report and Analysis

**DOI:** 10.1155/2020/4069431

**Published:** 2020-04-23

**Authors:** Marco Odorizzi, Maurice FitzGerald, Jorge Gonzalez, Dario Giunchi, Flurim Hamitaga, Vincenzo De Rosa

**Affiliations:** Paediatric and Orthopaedic Surgical Department, Istituto Pediatrico della Svizzera Italiana (IPSI), Bellinzona and Valli Regional Hospital, Ticino, Switzerland

## Abstract

Clavicular fractures are some of the most common bone injuries in the paediatric population, yet the rates of nonunion are very low under 18 years. To the best of our knowledge, posttraumatic nonunion of the clavicle in a paediatric population is rarely reported. We report the case of an 11-year-old girl who presented with a nondislocated fracture of the midshaft to the proximal third of the right clavicle. Initial conservative treatment by sling immobilization demonstrated radiologically confirmed healing at 3 months. However, at 1-year follow-up, she presented with painful nonunion. Diagnostic MRI and CT exams confirmed a pseudoarthrosis, requiring elective open reduction and internal fixation with the aid of an ipsilateral iliac crest bone graft.

## 1. Introduction

The clavicle is the most commonly fractured bone in children, representing 6% to 15% of all paediatric fractures [1]. The vast majority of these fractures involve the middle third, and the majority are ubiquitously treated conservatively, using sling immobilization and serial radiological check-ups [2, 3]. The reported incidence of nonunion in the paediatric population is extremely rare, in contrast to that known of the adult population, where the rate is from 8% to 14% [4]. A systematic review undertaken by Hughes et al. reported 21 paediatric cases of nonunion, the majority of which occurred after a displaced right-sided fracture and with typical presentation occurring at a distance of one year [5].

## 2. Case Report

**Figure 1:**
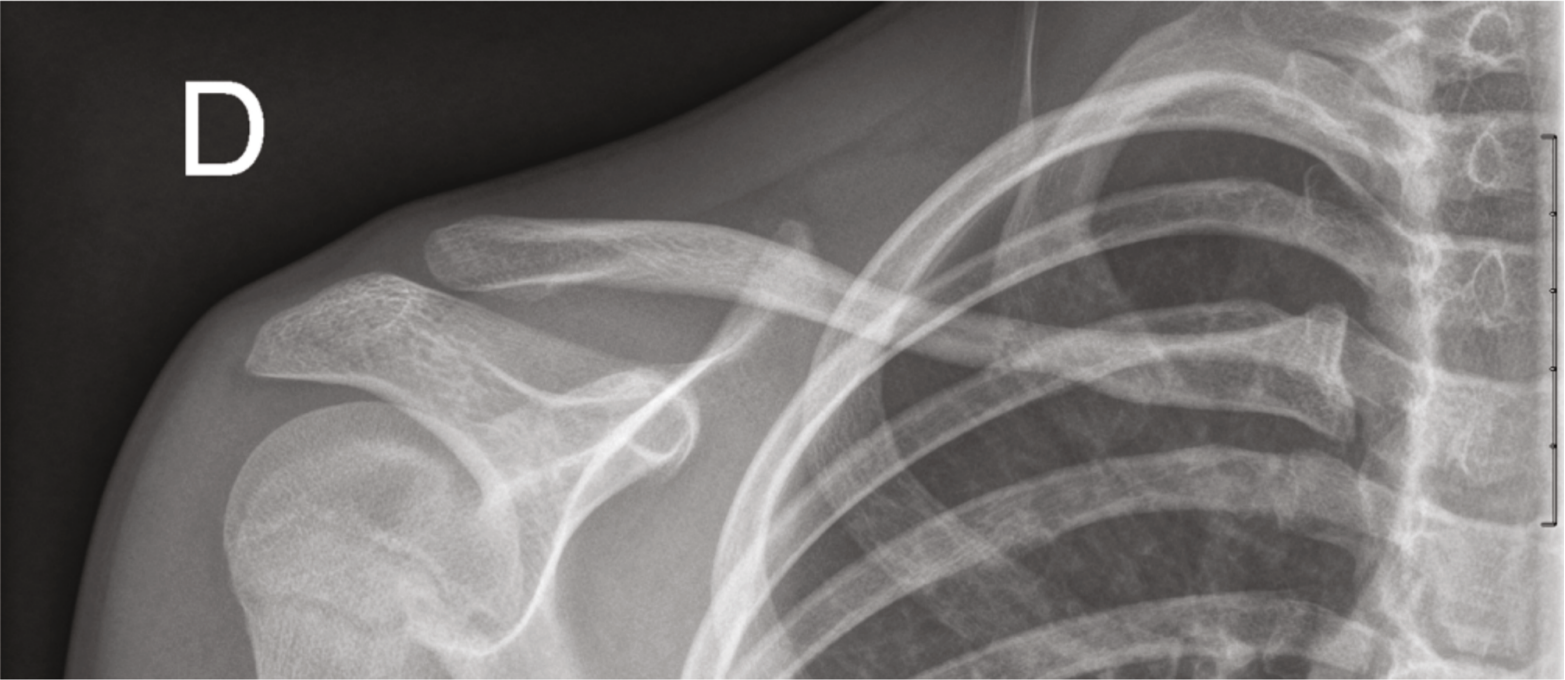
X-ray imaging showing the nondislocated fracture on the day of presentation to the paediatric A&E.

**Figure 2:**
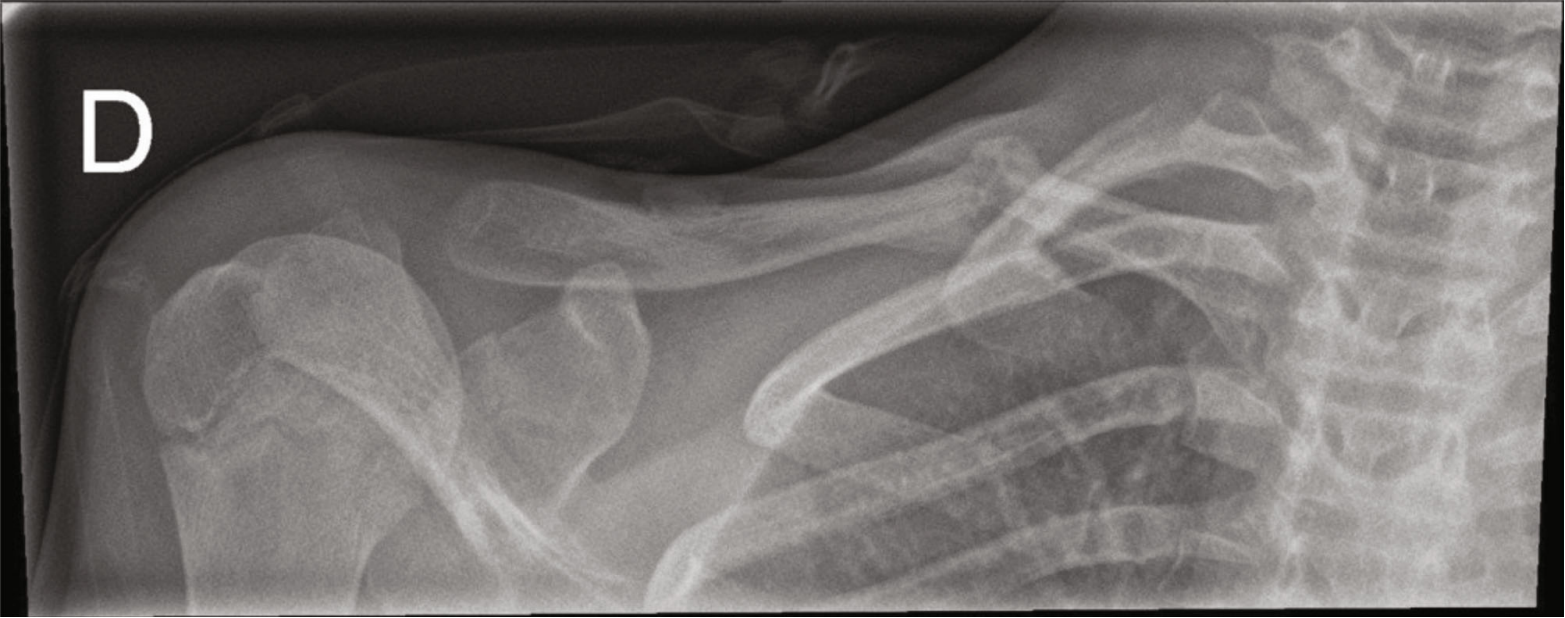
X-ray imaging showing callus formation after three months.

An 11-year-old girl presented to our A&E department after a fall on an outstretched arm with immediate pain experienced at the right shoulder. The X-ray demonstrated a nondisplaced fracture of the middle to proximal third of the right clavicle (Figure 1). There were no other injuries, and neurovascular examination of the upper extremity was normal. The fracture was managed conservatively with sling immobilization for 6 weeks, and on clinical follow-up, it appeared to be healing well. The patient had no pain nor functional limitation, with adequate progression of periosteal reossification and callus formation seen on radiological examination. The patient was an amateur junior javelin thrower and was restricted from all sporting activity for a total of 3 months and until there was radiographic confirmation of callus formation (Figure 2).

**Figure 3:**
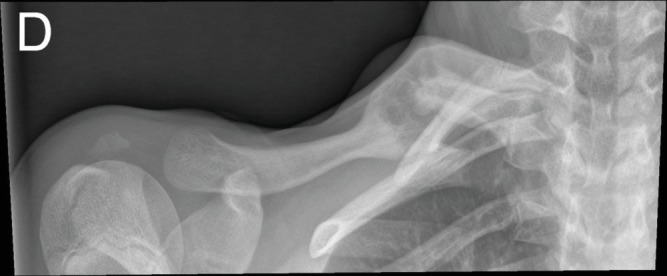
X-ray imaging showing partial callus formation, hypertrophic nonunion, and deformation at the boundary of the midshaft and proximal right clavicle.

The patient remained completely asymptomatic for 12 months, after which time she presented with new swelling at the callus midline, associated pain, and shoulder function loss, in the absence of additional trauma. The X-ray at 12 months confirmed the clinical suspicion of a pseudoarthrosis (Figure 3).

**Figure 4:**
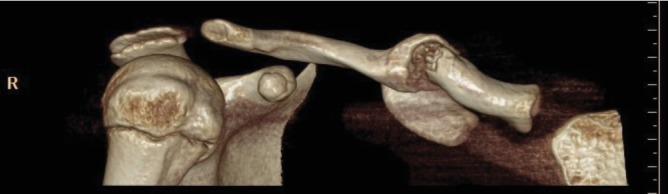
CT scan confirmed the diagnosis of the pseudoarthrosis.

Following a multidisciplinary discussion, we decided to investigate the suspected nonunion and need for surgical intervention using CT and MRI (as seen below). The initial topographic examination was important to validate the pseudoarthrosis, which clearly identified two areas of hypertrophy without signs of bridging. The MRI was useful in excluding an entrapment of surrounding articular structures and in evaluating the involvement of the neurovascular bundle with respect to the bone (Figure 4).

**Figure 5:**
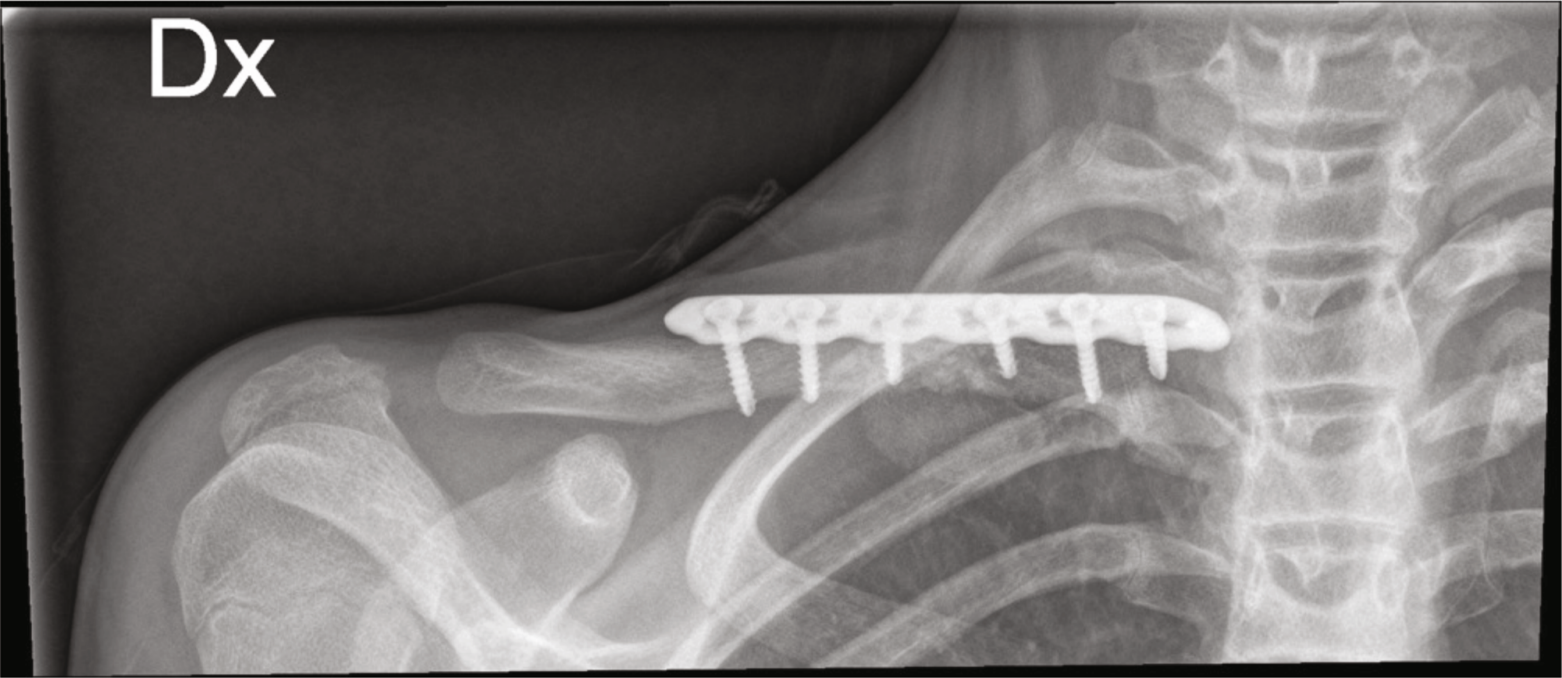
Postoperative X-rays.

Following a discussion with the patient's parents, we opted for a surgical approach with open reduction and internal fixation and iliac crest bone graft from the ipsilateral hip 3 × 3 × 1 cm. The nonunion was confirmed intraoperatively with debriding of the fracture site, with a loss of bone substance of 2 cm. The bone sample taken from the iliac crest was customised, molded, and inserted into the remaining gap. Following bone tissue transplant, the clavicle was reduced and internal fixation performed with a 3.5 mm seven-hole locking compression plate (LCP) (Synthes) in compression (Figure 5).

**Figure 6:**
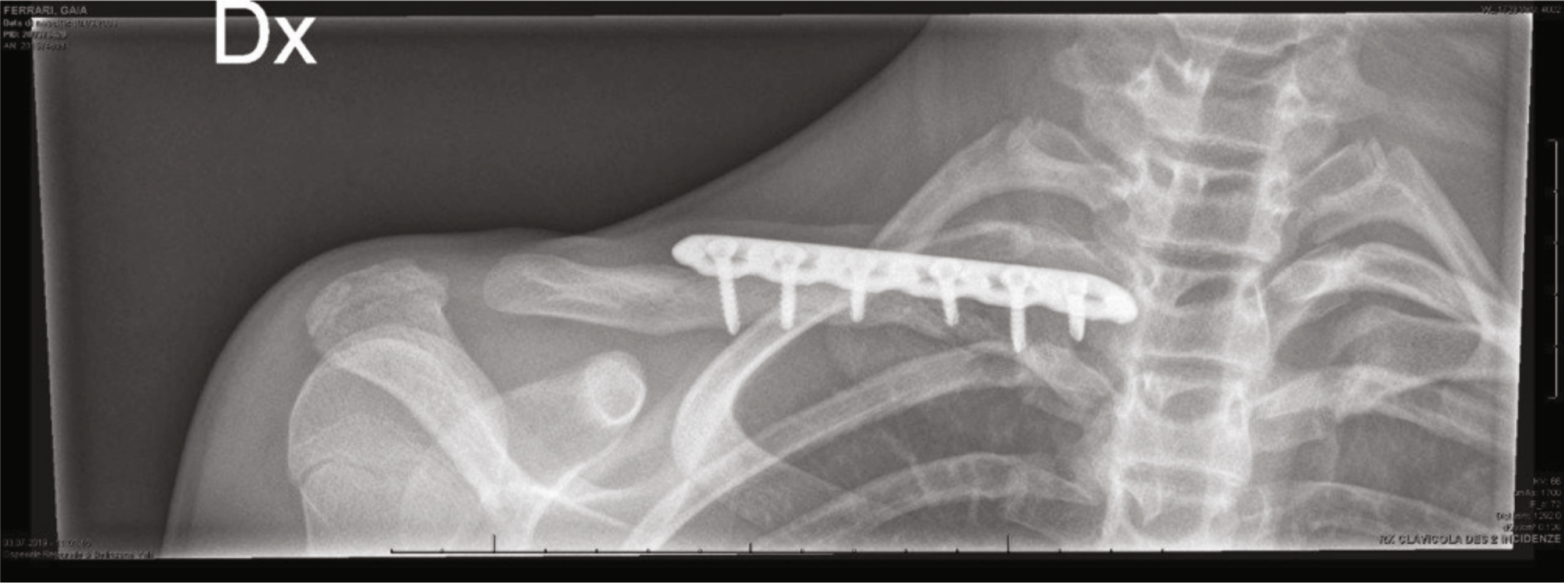
X-ray imaging at 1 year.

Following a normal postoperative course, clinical and radiological follow-up showed complete isometric anatomical healing at 1 year from the operation (Figure 5). The clinical examination showed no pain during shoulder movement, with a range of motion in antepulsion of 180°, abduction of 180°, internal rotation up to X thoracic vertebra, and external rotation of 80° (Figure 6).

## 3. Discussion

Clavicle fractures are common injuries in children and adults [1, 6], but generally the treatment is nonoperative with a sling immobilization and avoidance of high-risk activity such as contact sports for several weeks. The efficacy of this management is well described in the literature [2, 3, 7, 8]. Surgical treatment is very rarely performed with the most common indication being an exposed fracture, potential skin necrosis or perforation, and floating shoulder injuries [3]. Relative indications are a completely displaced fracture in high-performing adolescent athletes and/or for cosmetic purposes [2, 8–10]. In most cases, there is no difference in outcome between operative and nonoperative management [2, 11].

Paediatric clavicle fracture nonunion is a rare clinical occurrence [3, 7, 12, 13]. Even in large retrospective reviews of clavicle fracture in children, a precise nonunion rate is not yet known. For example, Kessler et al. [12] found no cases of nonunion, and Randsborg et al. [13] had only one case of nonunion out of 185 clavicle fractures. Completely displaced fractures and refractures of the ipsilateral clavicle may be predisposing factors for nonunion [3, 7] as described in the study by Strauss et al. [7]. Their findings demonstrated just one case of nonunion and three cases of delayed union out of 537 children. However, there is a limited number of reviews specifically evaluating paediatric nonunion.

A clinically relevant finding, that is of therapeutic importance, is to identify a nonunion from a congenital pseudoarthrosis of the clavicle from a posttraumatic nonunion [14]. These generally occur on the right side in females [15] and at birth present as a painless lump that may increase in size and become painful, without necessarily any history of trauma [14, 16]. Radiographic differences are well described by Owen and include that the medial half of the clavicle is large and protrudes forward and upwards, whereas the lateral half is situated below, pointing upwards and backwards and ending in a bulbous mass at the site of the pseudoarthrosis [16].

The prevalence of documented nonunion clavicle fractures result on the right-hand side. Indeed, Smith and William proposed a possible/probable connection of this predominance with the known predominance of congenitally acquired pseudoarthrosis, also occurring on the right-hand side [17]. The exact pathophysiological reason for such a connection is not further elaborated nor known, yet Smith and William identify the fact that many subclinical congenital pseudoarthrosis may not be identified or visible on X-ray nor by physical exam. This may lead to a lack of pathology recognition, thus predisposing such patients to a line of weakness in the case of trauma, leading to fracture at this pseudoarthritic site and subsequent misdiagnosis as a “posttraumatic nonunion” [5].

Another important aspect of case management is the necessity of performing serial imaging in asymptomatic children. Adamich et al. [18] demonstrated that the rate of detection does not change and therefore advocated against serial imaging. Instead, he advocated for an informative approach with patient or parent education of the possibility of nonunion and stressed the importance of “early presentation” as being critical as a clinical strategy, in order for early identification and prevention [18].

Paediatric posttraumatic clavicular nonunion is rare but remains a potential complication and therefore requires physician awareness and early identification. Though the case population is small and consensus is not unanimous as yet, it appears that clinical follow-up with serial X-ray imaging and radiation exposure, is a safe management option.

There is a possibility that posttraumatic pseudoarthrosis of the clavicle may have a congenital component, in that it is a population underdiagnosed until the time of presentation. However the discrimination between a “true” traumatic nonunion and a previously undiagnosed “congenital” nonunion may have little clinical impact on diagnosis, follow-up, and treatment strategies. Additionally, as in our case, when operative intervention is necessary, plate fixation and autologous graft usage proves an excellent therapeutic option.
